# The *E. coli* Effector Protein NleF Is a Caspase Inhibitor

**DOI:** 10.1371/journal.pone.0058937

**Published:** 2013-03-14

**Authors:** Sonja Blasche, Mario Mörtl, Holger Steuber, Gabriella Siszler, Shahista Nisa, Frank Schwarz, Inna Lavrik, Thomas M. A. Gronewold, Klaus Maskos, Michael S. Donnenberg, Dirk Ullmann, Peter Uetz, Manfred Kögl

**Affiliations:** 1 Genomics and Proteomics Core Facilities, German Cancer Research Center, Heidelberg, Germany; 2 Proteros biostructures GmbH, Martinsried, Germany; 3 School of Medicine, University of Maryland, Baltimore, Maryland, United States of America; 4 Division of Immunogenetics, German Cancer Research Center, Heidelberg, Germany; 5 Center for the Study of Biological Complexity, Virginia Commonwealth University, Richmond, Virginia, United States of America; 6 SAW Instruments GmbH, Bonn, Germany; University of Massachusetts Medical School, United States of America

## Abstract

Enterohemorrhagic and enteropathogenic *E. coli* (EHEC and EPEC) can cause severe and potentially life-threatening infections. Their pathogenicity is mediated by at least 40 effector proteins which they inject into their host cells by a type-III secretion system leading to the subversion of several cellular pathways. However, the molecular function of several effectors remains unknown, even though they contribute to virulence. Here we show that one of them, NleF, binds to caspase-4, -8, and -9 in yeast two-hybrid, LUMIER, and direct interaction assays. NleF inhibits the catalytic activity of the caspases *in vitro* and in cell lysate and prevents apoptosis in HeLa and Caco-2 cells. We have solved the crystal structure of the caspase-9/NleF complex which shows that NleF uses a novel mode of caspase inhibition, involving the insertion of the carboxy-terminus of NleF into the active site of the protease. In conformance with our structural model, mutagenized NleF with truncated or elongated carboxy-termini revealed a complete loss in caspase binding and apoptosis inhibition. Evasion of apoptosis helps pathogenic *E. coli* and other pathogens to take over the host cell by counteracting the cell’s ability to self-destruct upon infection. Recently, two other effector proteins, namely NleD and NleH, were shown to interfere with apoptosis. Even though NleF is not the only effector protein capable of apoptosis inhibition, direct inhibition of caspases by bacterial effectors has not been reported to date. Also unique so far is its mode of inhibition that resembles the one obtained for synthetic peptide-type inhibitors and as such deviates substantially from previously reported caspase-9 inhibitors such as the BIR3 domain of XIAP.

## Introduction

Pathogenic bacteria that cause infectious diarrhoea are a major health problem worldwide. Among them, EHEC and EPEC are especially virulent [1], [2]. Like many other pathogenic gram negative bacteria, they possess a type III secretion system (T3SS), a syringe-like apparatus composed of more than 20 proteins, which permits injection of bacterial effector proteins directly into mammalian host cells. EHEC strain O157:H7 (Sakai) encodes for 62 putative effectors, of which 39 are verifiably translocated into the host cell [3]. These proteins, many of which are shared by EPEC, are responsible for the subversion of cellular antagonistic responses and the establishment of an environment suitable for pathogen proliferation. The precise function of many of these effectors is unknown, even though some, including NleF, have been implicated in virulence [4].

Eukaryotic cells recognise invading pathogens by pattern recognition receptors, which stimulate the secretion of inflammatory mediators such as IL-1β, IL-6, TNFα and interferons to combat the infection. In some cases, the response concludes in the induction of apoptotic cell death to remove the invading pathogen along with its host cell [5]. Central to the execution of apoptosis are the caspases, a family of intracellular aspartate proteases [6] that become activated upon initiation of apoptosis. To counteract this response, many viruses express proteins which inhibit the induction of apoptosis [7]. Some anti-apoptotic viral proteins act as decoys for cellular pro-apoptotic factors, such as viral inhibitors of apoptosis (vIAPs) [8], whereas others, such as the baculoviral p35 protein, directly bind to caspases to inhibit their proteolytic activity [9], [10]. An example of a bacterial effector protein counteracting an apoptotic response has recently been reported: The EHEC and EPEC effector NleH inhibits apoptosis of host cells by targeting BI-1 (Bax inhibitor 1), a cellular inhibitor of the anti-apoptotic protein Bax [11]. However, direct inhibition of caspases by bacterial effectors has not been reported to date.

## Results

### NleF Binds to Caspases

**Figure 1:**
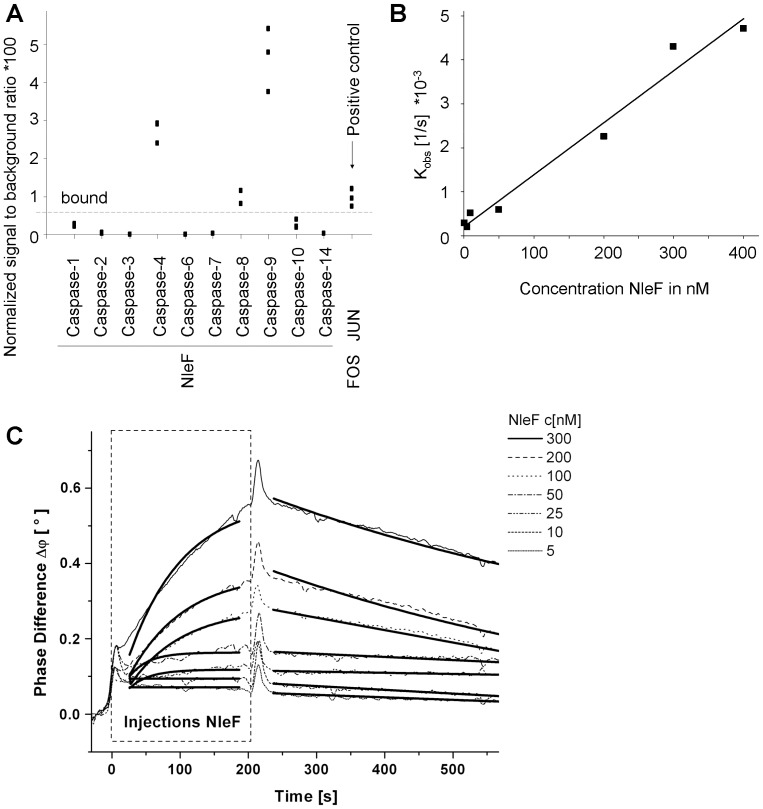
NleF binds caspases -4, -8 and -9. **A.** Binding of NleF to caspases-4, -8 and -9 using luciferase-NleF and protein-A-caspase fusions (LUMIER assays^34^). Interaction strengths are expressed as signal to background ratios using binding to protein A as a negative control. The interaction of JUN to FOS serves as a positive control. Squares indicate individual measurements. **B,C.** The affinity constant (K_d_) of NleF binding to immobilized caspase-9 as measured by surface acoustic wave (SAW) technology is ∼39 nM. The K_d_ is defined as follows: K_d_ = K_off_/K_on_ = y-intercept/slope (**C)**, SAW overlay plot of NleF injections. NleF concentrations are indicated in the legend. The two bold curves added to each NleF measurement (on the left and right of the peak) represent the optimal K_obs_ and K_off_ curves.

In a systematic effort to elucidate the cellular targets of EHEC effector proteins, we identified caspase-9 as a binding partner of NleF. Caspase-9 was the predominant hit identified in screens of NleF against amino- and carboxy-terminally tagged yeast two-hybrid libraries (see **Methods** for details). LUMIER assays with caspase-9 and NleF in HEK-293T cells confirmed the interaction**.** A test for interaction of NleF with caspases-1, -2, -3, -4, -6, -7, -8, -9, -10 and -14 led to the identification of caspase-4 and caspase-8 as additional binding partners (**Fig. 1A**). We determined the dissociation constant of the Caspase9-NleF interaction by surface acoustic wave resonance (SAW) to be ∼39 nM, a value which is well in the range of known viral inhibitors of apoptosis [7], indicating biological significance (**Fig. 1B, C**, **Table S1**).

### NleF Inhibits Human Caspases-4, -8, and -9

**Figure 2:**
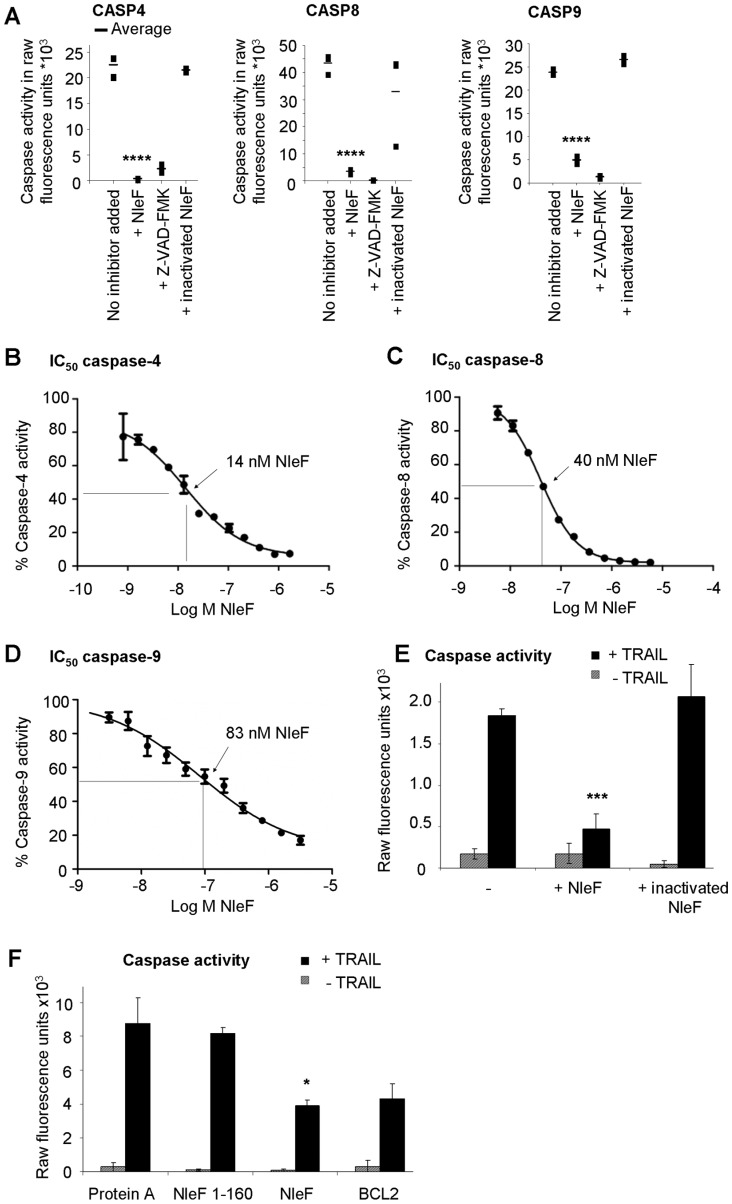
NleF inhibits caspases *in vitro* and in in cells. **A.** *In vitro* inhibition of caspase-4 (1 U), caspase-8 (1 U) and caspase-9 (1 U) by NleF (1.5 µg) (1.5 µg/unit caspase), the inhibitor Z-VAD-fluoromethylketone (Z-VAD-FMK, 20 µM), and proteinase K-inactivated NleF (1.5 µg). **B-D.** Dose-dependent inhibition of purified caspase-4, -8, and -9 by NleF (logarithmic concentration indicated in the X axis): **B.** Caspase-4 (200 nM). **C.** caspase-8 (68 nM). **D.** caspase-9 (1.1 µM). **E.** Recombinant NleF inhibits caspase-9 activity in lysates of apoptotic cells. HeLa cells were induced to enter apoptosis by treatment with TRAIL (25 ng/ml) for four hours (black bars) or left untreated (striped bars) before preparation of lysates and measurement of cellular caspase-9 activity in the absence or presence of an abundant amount of purified NleF. **F.** Caspase-9 activity in apoptotic HeLa cell extracts expressing Protein A, an inactive fragment of NleF (amino acids 1–160), wild type NleF or BCL2, respectively. Significance was determined using the two-tailed unpaired Student’s t-test. *p<0.05; **p<0.01; ***p<0.001; ****p<0.0001.

Binding of NleF to purified caspase-4, -8, and -9 resulted in potent inhibition of all three caspases (**Fig. 2A** **, Fig. S1**). The inhibitory activity of NleF was lost after proteinase K treatment, excluding a contribution from non-proteinaceous contaminants. To accesss the inhibition of caspase activity in vitro by NleF in a dose-dependent way serial dilutions of NleF were tested for their effects on caspase activity and the IC-50 of NleF for inhibition of caspase-4, -8 and -9 were determined to be 14 nM, 40 nM and 83 nM (**Fig. 2B–D**). NleF, but not proteolytically inactivated NleF, significantly inhibited caspase activity in extracts of HeLa cells induced to enter apoptosis with TRAIL (Tumor Necrosis Factor Related Apoptosis Inducing Ligand; **Fig. 2E**). A reduction in caspase-9 activity was also observed in lysates of TRAIL-treated HeLa cells expressing the apoptosis-inhibitory protein BCL2 or wild type NleF, respectively, whereas expression of an interaction-deficient mutant of NleF (NleF 1–160, see **Fig. S2**) had no effect on caspase-9 activity (**Fig. 2F**).

### NleF Prevents the Induction of Apoptosis

**Figure 3:**
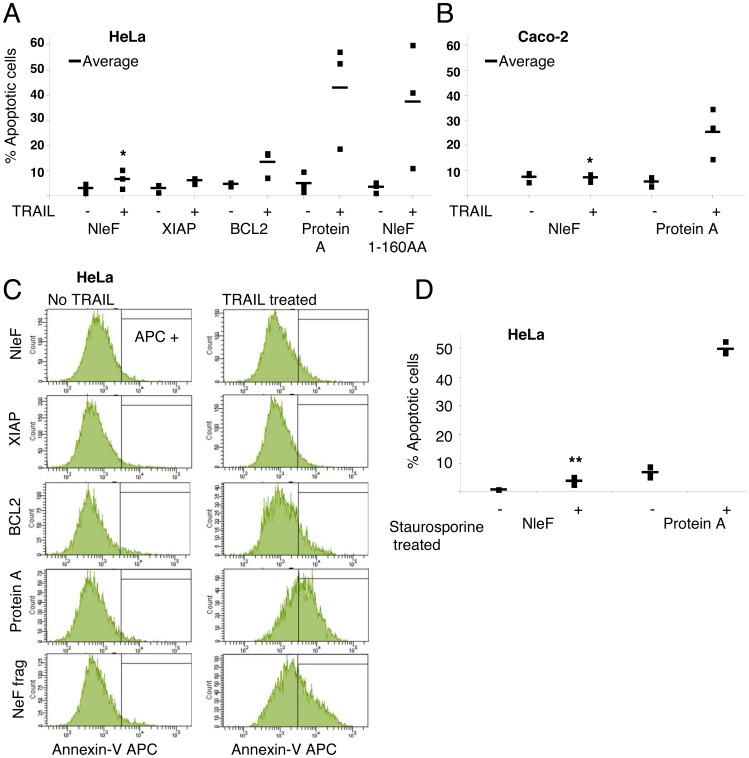
NleF inhibits apoptosis induced by TRAIL and staurosporine. **A–C.** Percentage of apoptotic HeLa and Caco-2 cells in TRAIL (25 ng/ml) treated (+) and untreated (−) samples. **A.** HeLa cells expressing YFP-fusions of wild type NleF, XIAP and BCL2, respectively, exhibited decreased apoptosis in comparison to cells expressing Protein A or an inactive NleF fragment (amino acids 1–160) after TRAIL treatment. Squares: percentage of apoptotic cells (three independent experiments); bars: average. **B.** Caco-2 cells expressing NleF and Protein A, respectively. Significance (t-test): *P<0.05. **C.** Representative FACS counts of HeLa cells expressing the indicated YFP-tagged constructs. Cells that stained positive for annexin V- allophycocyanine (APC+) but negative for propidium iodide were counted as apoptotic cells. **D.** Percentage of apoptotic HeLa cells in staurosporine treated (+) and untreated (−) samples expressing NleF and Protein A, respectively.

To test if caspase inhibition by NleF can prevent the induction of apoptosis, we transiently expressed NleF as well as known inhibitors of apoptosis in HeLa cells prior to induction of apoptosis with TRAIL. The fraction of cells entering TRAIL-induced apoptosis as assessed by annexin V staining was markedly reduced in HeLa cells expressing YFP-tagged NleF (6.5%) compared to cells expressing a Protein A control construct (42.8%). The magnitude of this effect was comparable to the effect of known inhibitors of apoptosis, such as XIAP (6.1%) and BCL2 (13.4%, **Fig. 3A, C** **and Fig. S3**). Experiments in Caco-2 colon carcinoma cells confirmed these observations (7.0% versus 25.2% induction for NleF- and Protein A- expressing cells, respectively; **Fig. 3B**). Cells expressing Protein A or an inactive fragment of NleF (amino acids 1–160) showed no significant reduction in apoptosis (**Fig. 3B**).

TRAIL triggers apoptosis through the extrinsic pathway, which is executed via caspase-8 as the initiator caspase (**Fig. S1**). However, caspase-9 has been shown to be essential for execution of TRAIL-induced apoptosis of many somatic cells, including HeLa and Caco-2. To test if the anti-apoptotic effect of NleF is of comparable strength when caspase-9 is the only initiator caspase involved, we repeated the experiments using staurosporine as an inducer of intrinsic apoptosis. Only 3.7% of HeLa cells expressing NleF underwent apoptosis, as compared to 49.9% expressing Protein A (**Fig. 3D**), indicating that NleF is competent to inhibit caspase-9-dependent apoptosis in HeLa cells.

### Structural Basis of Caspase Inhibition by NleF

**Figure 4:**
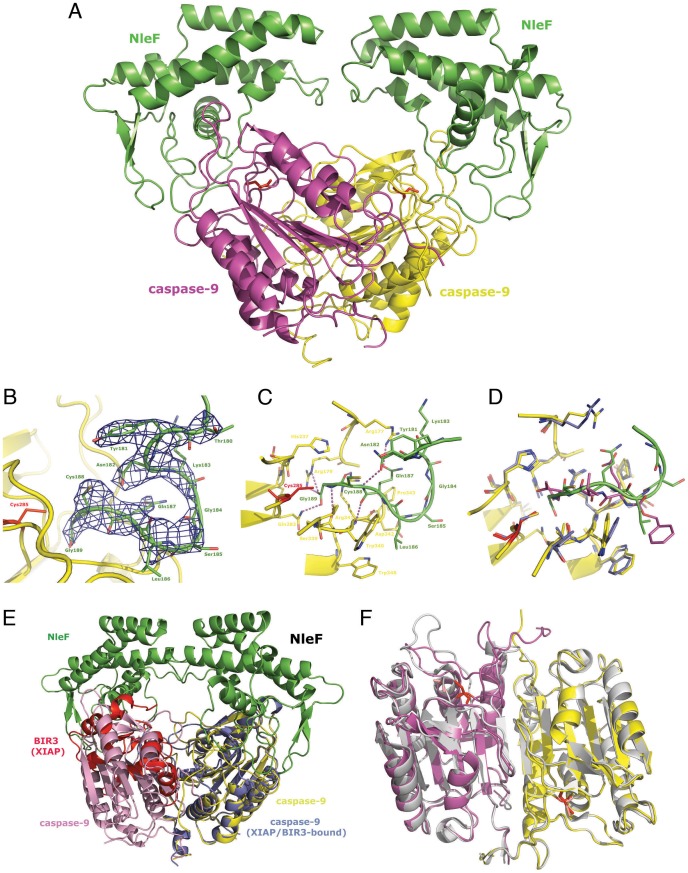
Crystal structure of Caspase-9 with bound NleF. **A.** The caspase-9 protease monomers are shown in magenta and yellow, the active site cysteines (Cys285) are shown as red sticks for improved special orientation of the catalytic site. The NleF effector proteins attached to the caspase active sites are shown in green, as labeled in the figure. **B.** The C-terminal residues of NleF are anchoring the effector protein to the specificity pockets of the protease. The Fo-Fc omit electron density contoured at 2.5 σ is shown in blue for a representative part of the involved C-terminal NleF residues. **C.** Detailed binding mode of the NleF C-terminus to the caspase-9 active site. Involved amino acids are shown as green and yellow sticks, hydrogen bonds are depicted as magenta dashed lines. **D.** Superposition of the NleF(green)-bound caspase-9 (yellow) with the peptide inhibitor Z-EVD-Dcbmk (magenta) bound to the caspase-9 monomer (blue) captured in active state (PDB entry 1JXQ). While the conformation of caspase residues is virtually identical, slight differences in the mode of interaction of the bound inhibitors are e.g. observed for the Asp/Gly-P1 moiety, where the Asp of Z-EVD-Dcbmk adopts a more favourable geometry for efficient salt bridge formation to Arg179. **E.** Superimposition of the NleF-bound caspase-9 dimer presented in this study and the previously described caspase-9 crystal structure (light blue) in complex with the BIR3 domain of the XIAP inhibitor shown as red cartoon (PDB entry 1NW9). While the latter interferes with the formation of a productive caspase dimer, NleF establishes its inhibitory activity by means of a fundamentally different mechanism of active site targeting. **F.** Superimposition of the caspase-9 dimer obtained for the NleF-inhibited form (yellow and magenta as in **A.,** both NleF molecules omitted for clarity) and the dimer of 1JXQ (grey cartoon). While the type of dimer formation and the overall protein conformation is well conserved between the two dimers, deviations arise mainly from the fact that one of the two 1JXQ monomers (left) corresponds to a non-productive, inactive conformation while in the NleF-inhibited dimer, both monomers adopt an active state with well-formed specificity pockets.

To determine how NleF inhibits caspase-9, we co-crystallized the protein complex and determined its structure at a resolution of 3.49 Å (**Table S2**). Bound to NleF, caspase-9 shows the typical fold of a mostly parallel six-stranded β-sheet sandwiched between two layers of α-helices and forms a dimer arranged in a similar geometry to that described for unbound caspase-9 [12]. NleF folds into a four-helix bundle connected by a two-stranded β-sheet between α-helices 2 and 3 as well as extended loop regions between the α-helices. A Dali search [13] for closest structural neighbours of NleF produces results with a Z-score of 6.4 or lower, indicative of only limited topological similarity to PDB-deposited protein structures. Each caspase monomer bears one NleF molecule bound via multiple interactions of the substrate recognition site of the caspase to the effector protein (**Fig. 4A** **, Fig. S4**). The interaction interface between caspase-9 and NleF comprises 902 and 1017Å^2^ of the two proteins, respectively. Despite moderate crystallographic resolution, the omit Fo-Fc difference electron density map allows for an unambiguous interpretation of the NleF interaction mode (**Fig. 4B**). In contrast to the published structure of a caspase-9 dimer, with one active and one inactivated protease monomer [12] per asymmetric unit, in the NleF-bound state, both caspase molecules are trapped in a binding-competent (but proteolytically inactive) state with well-established subpockets. The C-terminal amino acids Gly189, Cys188, Gln187 and Leu186 of NleF anchor the effector protein to the S1, S2, S3, and S4 pocket of the protease, respectively (**Fig. 4C**). The S1 pocket that typically recognizes the P1 aspartate residue harbours Gly189 of NleF while the C-terminal main chain carboxylate group of this moiety partly mimics the interactions of the Asp side chain carboxylate. The side chains of Arg179 and Arg341 are involved in electrostatic interactions/salt bridges to NleF Gly189, whereas Cys188 occupies the S2 pocket of the protease, which mainly consists of the space between the aromatic side chains of Trp340 and His237. This pocket typically tolerates only amino acid side chains of small and aliphatic nature such as Ala and Val [14]. NleF Gln187 occupies the protease S3 pocket and mainly interacts with the Arg341 peptide NH group via its main chain carbonyl oxygen and forms a charge-assisted H-bond from the Gln side chain carbonyl to the positively charged Arg177 side chain. The S4 pocket is occupied by NleF Leu186 which establishes van der Waals contacts to the aromatic side chains of Trp340 and Trp348 of caspase-9.

The inhibition mode of NleF to some extent resembles the one obtained for synthetic peptide-type inhibitors such as Z-EVD-Dcbmk^12^, where the modified Asp, Val and Glu occupy the caspase-9 S1, S2, and S3 pockets while the benzyloxy-carbonyl moiety is partly solvent-exposed and occupies the S4 pocket.However, the latter inhibitor seems to establish a more efficient salt bridge between its Asp to Arg179 and Arg341, which becomes only partly mimicked by the NleF-Gly189 C-terminus (**Fig. 4D**). This inhibition mode observed for NleF deviates substantially from previously reported caspase-9 inhibitors such as the BIR3 domain of XIAP [15], which occupies the dimerisation interface of two caspase-9 molecules thereby inducing a catalytically non-productive conformation of the protease (**Fig. 4E**). We have not been able to detect dimerisation of NleF by itself (**Fig. S5**). Interestingly, despite the overall structure of the caspase dimer and the type of dimerisation, caspase-9 structures share high similarities for the NleF-bound caspase presented in this contribution and the dimer inhibited by Z-EVD-Dcbmk^12^ (**Fig. 4F**). However, in the present complex both caspase monomers are captured in a productive state with well established specificity pockets. Thus, with respect to the caspases, the two crystal structures mainly differ in the conformation of the loop beginning with Ala284 bearing the active site Cys285, which becomes undefined after Gly287 in the previously reported structure and is defined to Ser298 in the NleF-bound complex. In addition, consistent with this active conformation the loop region Ser332–Ser347 between strand 7 and helix E deviates between the two structures and contributes in active state to the dimer interface (**Fig. 4F**).

### Effect of NleF in an Infection Model

**Figure 5:**
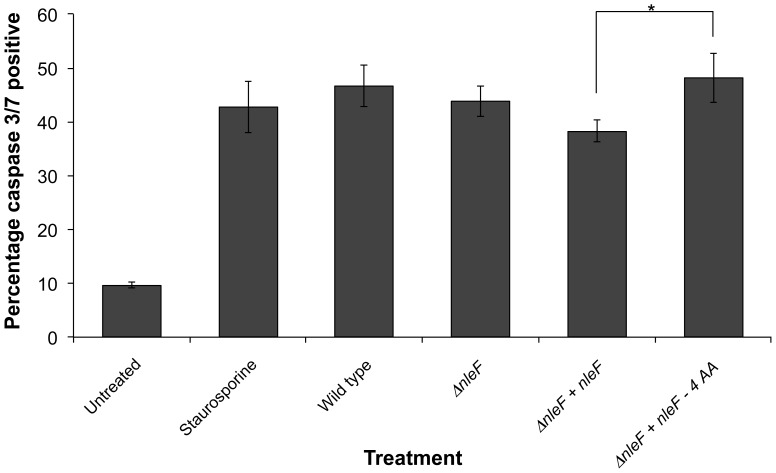
Caspase 3/7 activity in infected HeLa cells. HeLa cells were infected with wild type EPEC strain E2348/69, Δ*nle*F deletion mutant, or Δ*nle*F complemented in single copy with *nle*F variants for 2.5 to 3 hours. Treatment of HeLa cells with staurosporine (5 µM) was used as a positive control. There was no significant difference between caspase activity upon infection with the wild type strain and the Δ*nle*F deletion mutant, however the deletion mutant complemented with the wild type allele consistently induced significantly less caspase 3/7 activity than the deletion mutant complemented with the allele lacking the last 4 codons. Significance was determined using the two-tailed Wilcoxon test *p<0.02.

To determine whether NleF can inhibit apoptosis when injected into cells via the type III secretion apparatus, HeLa cells were infected with enteropathogenic *E. coli* (EPEC), which shares the T3SS and many of the effectors, including an identical NleF, with EHEC. To ensure that the effect of NleF on caspases-4, -8 and -9 were captured in the infection model, activation of the downstream caspase-3/7 was assessed to measure apoptosis. We detected no significant difference between wild type EPEC and an *nleF* mutant in the ability to induce caspase 3/7 activation **(** **Fig. 5** **)**, indicating that under these conditions and in the context of the full repertoire of effector proteins NleF does not play a dominant role. However, we did find a significant difference in apoptosis between the cell lines infected with the *nleF* mutant complemented in single copy with the wild type *nleF* allele and the mutant similarly complemented with a caspase interaction-deficient *nleF* allele (NleF with the last four amino acids deleted, see also below). The ability to detect this difference in the complemented strains can be attributed to the fact that the complementation strains had higher levels of expression than the WT strain (**Fig. S6**). This slightly increased expression of NleF resulted in less caspase 3/7 cleavage in the mutant complemented with the wild type allele in comparison to the other strains tested (**Fig.** **5**).

### Validation of the Structural Model

**Figure 6:**
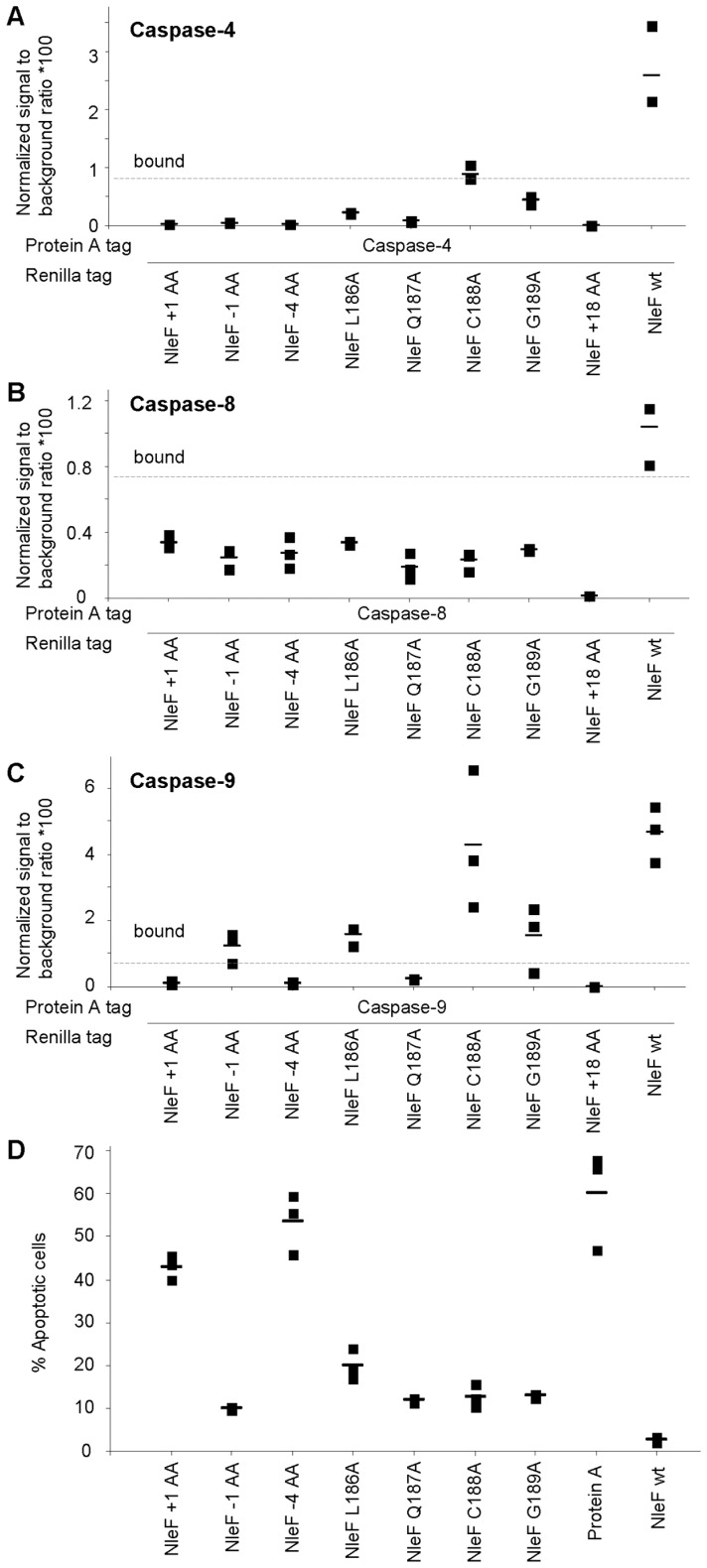
Caspase binding and apoptosis inhibition by modified versions of NleF. **A–C.** Caspase-9-binding of different versions of NleF assessed by LUMIER assays. **A.** Caspase-4, **B.** caspase-8, **C.** caspase-9. **D.** Percentage of apoptotic HeLa cells expressing different versions of NleF after induction with staurosporine. NleF +1: NleF with an additional C-terminal alanine; NleF -1 and NleF -4: NleF with the terminal 1 and 4 amino acids removed, respectively; NleF L186A, NleF Q187A, NleF C188A and NleF G189A: NleF versions with indicated amino acids substituted by alanine; NleF +18: NleF with additional 18 amino acids.

To test the predictions of this structural model, we generated versions of NleF with truncated or elongated C-termini, as well as individual mutations of the carboxy-terminal four amino acids to alanine. The effect of these alterations on binding to caspases and inhibition of apoptosis is depicted in **Fig. 6**. Deletion of the last four amino acids of the NleF carboxy-terminus or extension by a single amino acid reduced caspase binding and anti-apoptotic activity to background levels (**Fig. 6A–D**). Deletion or mutation of single amino acids also reduced the anti-apoptotic activity, but did not completely abolish it. In accordance with this effect, interaction signals above background were still detectable for the single amino acid deletion variant of NleF and caspase-9, and the Cys188Ala mutation variant and caspase-4 and -9. These data strongly support the conclusion drawn from the crystallographic analysis, showing that caspase inhibition by NleF occurs by a novel type of inhibitory interaction of the carboxy-terminus of NleF and the active site of the caspase.

## Discussion

The elimination of infected cells via apoptosis is an evolutionary conserved defence mechanism of multicellular organisms that is commonly used against viruses, pathogenic bacteria and other parasites. To counteract this mechanism, many pathogens, such as cytomegaloviruses, *Mycobacterium tuberculosis* and *Toxoplasma gondii* have evolved mechanisms to prevent apoptosis of their host cells [5], [16], [17]. The need for this countermeasure may be all the more crucial to pathogenic *E. coli* as the bundle-forming pili and effectors expressed by EPEC and/or EHEC, e.g. EspF and Map, have been demonstrated to induce apoptosis in host cells [18], [19], [20], [21], [22]. Despite the presence of these proapoptotic factors, a decrease in normal apoptotic rates was observed upon infection of rabbits with a related REPEC O103 strain [23]. EPEC and EHEC inject into host cells a large number of effector proteins that can influence apoptosis including pro- and anti-apoptotic proteins. In the context of this large repertoire of effectors, loss of NleF did not significantly change EPEC induced apoptosis, a result consistent with the low levels of NleF expressed [4]. However, complementation of the *nleF* deletion mutant with wild type and mutant *nleF* alleles resulted in slightly higher levels of expression, even though the genes were inserted into the chromosome in single copy. Hence, NleF indeed plays a role in inhibiting apoptosis as expression of NleF variants unable to bind caspase-9 resulted in increased levels of caspase 3/7 cleavage.

With caspase-9, NleF targets a bottleneck in the intrinsic apoptotic pathway and is able to counteract apoptosis even at a point when pro-apoptotic proteins have already been released from mitochondria (**Fig. S1)**. Most somatic cells, including enterocytes (the relevant host cells for EHEC and EPEC), depend on mitochondrially induced caspase-9 activation for induction of apoptosis [24], [25], [26]. In addition to caspase-9, NleF binds to and inhibits caspase-8, the initiator caspase triggering the extrinsic apoptotic pathway [27]. NleF also blocks caspase-4, which has recently been reported to be required for the activation of the inflammasome [28]. Inhibition of caspase-4 by NleF might thus also inhibit the inflammation response. Targeting these caspases allows NleF to block apoptotic as well as immune response signals before they reach the downstream executioner caspases 3/7 and thus prevent the host cells from erasing themselves and thereby the invading pathogen. We conclude that NleF’s interference with these cascades contributes to the preservation of the biological niche of EHEC, EPEC and possibly other pathogens that express homologous effector proteins.

## Materials and Methods

### Oligonucleotides and Bacterial Strains

ECs1815 (*nle*F) was cloned from *E. coli* O157:H7 str. Sakai using the Gateway® Technology (Invitrogen). Two sets of primers were used; first the gene-specific primers (ECs1815 forw: 5′- AGG CTc cacc ATG TTA CCA ACA AGT GGT TC-3′ and ECs1815 rev: 5′- C TGG GTG GAT TCA TCC ACA TTG TAA AGA TCC TTT GTT G-3′), and in a second PCR step the attB primers (attB1∶5′- G GGG ACA AGT TTG TAC AAA AAA GCA GGC TCC ACC ATG-3′ and attB2∶5′-GGG GAC CAC TTT GTA CAA GAA AGC TGG GTG GAT TCA-3′) for the attachment of the attB sites. Cloning and PCR were performed according to the manufacturer’s instructions. The resulting *nle*F PCR product was cloned via a Gateway® BP reaction in the entry vector pDONR221. Starting from the latter, *nle*F was shuttled into pcDNA3-Rluc-GW, pTREX-dest30-ntPrA, pGBKCg, pGBT9-GW and pdEYFP-C1amp [29], [30], [31], [32].

### Cell Lines, Transfection and Apoptosis Induction

Transient transfection of HeLa, Caco-2 and HEK293T cells was done using Lipofectamine (Invitrogen). The procedure was performed as recommended by the manufacturer. Apoptosis induction was achieved by adding 25 ng/ml SuperKillerTrail (ENZO Life Sciences) or 5 µM staurosporine (Roche Diagnostics) 6 h in advance of flow cytometric analysis or 4 h before lysis for the determination of caspase-9 activity in cell lysates. Caco-2 cells were obtained from ATCC (HTB-37). HEK293 were obtained from DSMZ (No. ACC 305).

### Yeast Two-hybrid Pool Screening

Yeast two-hybrid (Y2H) pool screening was done as described in Albers et al. [32]. Human libraries used in this study are the MGC ORF collection and the universal human cDNA library (Clontech 638874). The human ORF collection is a normalized human full length clone collection that originates from the Mammalian Gene Collection (MGC) [33]. The whole collection was obtained as an entry clone collection and subsequently shuttled to different expression vectors via site specific recombination [34] using the Gateway® Technology (Invitrogen). The vectors used for ORF library generation were pGAD424-GW and pGADCg, two Y2H vectors harboring N- and C-terminal fusions to the GAL4 activation domain, respectively.

### Lumier Assay (LUminescence-based Mammalian IntERactome Mapping) [35]

The method was performed as previously described [29]. Proteins were transiently expressed in HEK-293 cells as hybrid proteins with the *Staphylococcus aureus* protein A tag or with the *Renilla reniformis* luciferase ORF fused to their amino termini. 20 ng of each expression construct were transfected into 10,000 HEK293 cells using 0.05 µl of lipofectamine 2000 (Invitrogen) in 96 well plates. After 40 hours, medium was removed and cells were lysed on ice in 10 µl of ice-cold lysis buffer (20 mM Tris pH 7.5, 250 mM NaCl, 1% TritonX-100, 10 mM EDTA, 10 mM DTT, Protease Inhibitor Cocktail (Roche), Phosphatase Inhibitor Cocktail (Roche), Benzonase (Novagen) 0,0125 units per µl final concentration) containing sheep-anti-rabbit IgG-coated magnetic beads (Invitrogen, Dynabeads M280, 2 mg/ml final concentration). Lysates were incubated on ice for 15 minutes. 100 µl of washing buffer (PBS, 1 mM DTT) were added per well, and 10% of the diluted lysate was removed to determine the luciferase activity present in each sample before washing. The rest of the sample was washed 6 times in washing buffer in a Tecan Hydroflex plate washer. Luciferase activity was measured in the lysate as well as in washed beads. Negative controls had been transfected with the plasmid expressing the luciferase fusion protein and a vector expressing a dimer of protein A. For each sample, four values were measured: the luciferase present in 10% of the sample before washing (“input”), the luciferase activity present on the beads after washing (“bound”), and the same values for the negative controls (“input nc”, and “bound nc”). Normalised signal to background ratios are calculated as follows: Generate “signals normalised for expression levels” by calculating “bound”/“input” for both the interaction test (X-protein A+ Y-luciferase) and the negative control (protein A alone+Y luciferase [“bound nc”/“input nc”]). Divide the “signals normalised for expression levels” of the interaction test by the “signals normalised for expression levels” of negative control to derive signal to background ratios.

### Determination of the K_d_ for Caspase-9/NleF Interaction

Binding kinetic and affinity was analysed on sam®5 BLUE and sam®5 GREEN instruments (SAW Instruments GmbH, Bonn, Germany). Both instruments use the "surface acoustic wave"-principle for label-free analysis of molecular binding in a flow-through system as described in Perpeet et al. (2006) [36].

The K_d_ of NleF binding to immobilized caspase-9 was determined using the OriginPro 8.5-based FitMaster. A model derived from 1∶1 binding was applied. BSA and Protein A references were subtracted for obtained data, respectively.

### Detection of Caspase Activity in Cell Lysates

Caspase activity in lysates of HeLa cells was determined using the caspase-9 fluorometric assay kit APOPCYTO (MBL). Basic activity that could not be inhibited by the caspase inhibitor LEHD-FMK was substracted from caspase value. Caspase-9 inhibition in cell lysates by purified NleF was performed using 2.5 µg NleF per 25 µl HeLa cell lysate prepared from 25,000 cells.

### Measurement of Caspase Activity *in vitro*

Caspase-9 was purified as described below with specific activities around 1000 units per mg caspase. The proteolytic activity of purified caspase-9 was determined via Caspase-Glo® 9 (Promega). This caspase was used for all experiments using purified caspase-9 except **Fig.** **2** panel A, in which caspase-9 from the Abnova caspase-9 assay kit (KA0763) had been used. Caspase-4, -8 and -9 activities were measured using caspase-4, -8 and -9 inhibitor screening kits from Abnova (CASP4: KA0747, CASP8: KA0758 and CASP9: KA0763). Purified caspases were provided as a part of above mentioned kits. Specific activities were 1111 units for caspase-4/mg, 2500 units/mg for caspase-8, and 400 units/mg for caspase-9. For NleF inhibition, 1.5 µg NleF was added to 1 unit of active caspase. IC-50 of caspase-4, -8, and -9 was determined using 200, 68, and 1100 nM of caspase, respectively, using the Abnova kits for caspase-4 and -8 and the Caspase-Glo® 9 kit (Promega) for caspase-9. Concentration of caspase active sites was determined by titration with Z-VAD-FMK (MP Biomedicals). Fluorescence and luminescence were detected using the Tecan Infinite® 200 PRO series micro plate reader. Each experiment was done at least in triplicate. IC50 values were derived from two independent data sets done in triplicate using GraphPad Prism Software.

### Flow Cytometry Analysis

HeLa or Caco-2 cells were transfected with pdEYFP-C1amp containing YFP-fusions of BCL2, Protein A, XIAP, *nle*F and an *nle*F fragment (amino acids 1–160). Apoptosis was induced for 6 h, 48 hours after transfection. Subsequently, the cells were harvested and stained with propidium iodide (PI) and annexin-V-allophycocyanine (APC) (both BD Biosciences Pharmingen) for detection of necrotic and apoptotic cells, respectively. PI and Annexin-V-APC staining was performed following the manufacturer’s instructions. Flow cytometry was performed using the BD FACSCanto II flow cytometer with a 488 nm laser. PI, Annexin-APC and YFP were measured with a 670 nm long pass filter, a 660/20 nm and a 530/30 nm band pass filter, respectively. Blots were analyzed using the FACSDiva software (BD Biosciences).

### Protein Purification, Crystallisation and Structure Determination

The catalytic domain of caspase-9 (residues 140–416) and NleF (1–189) were co-expressed as His6- and GST-tagged fusion proteins, respectively, and co-purified via affinity chromatography against the GST-tag. After proteolytic cleavage of the GST-tag by enterokinase, the complex was purified by a second affinity chromatography step (Ni-NTA) against the NleF-fused His6 tag. Finally, a gel filtration chromatography step was performed to obtain the purified caspase-9-NleF complex. The protein complex was concentrated to 20 mg/ml in the final protein buffer (25 mM Hepes pH 7.5, 250 mM NaCl), and subjected to initial screening for appropriate crystallographic conditions. Initial crystals were obtained from a reservoir condition containing 0.75 M potassium-sodium tartrate. Data were collected under cryo-conditions at the Swiss Light Source, Villigen, Switzerland, using 15% (V/V) ethylene glycol in reservoir solution as cryoprotectant. Data were processed using XDS [37], phases were obtained by molecular replacement with Phaser [38] using the monomeric caspase-9 from the publicly available protease dimer [12] as a search model. Manual model building and refinement was done using Coot [39] and Refmac5 [40], respectively. Data collection and refinement statistics are listed in **Table S2**. Coordinates and reflection data have been deposited with the Protein Data Bank (www.pdb.org). The structural model has been validated via the Molprobity [41] server yielding an overall score of 1.02 (100^th^ percentile) and a Clashscore of 0.25 (100^th^ percentile), emphasizing the excellent quality of the structural model.

### Generation of NleF Mutations for Binding Assays

Site-directed mutagenesis of the NleF C-terminus was done using modified reverse primers along with the ECs1815 forward primer. For the NleF addition/deletion mutants, namely NleF+1 (plus one additional alanine), NleF-1 (Gly189 removed), NleF-4 (the last four amino acids removed), NleF 1–145 and NleF 1–160, the reverse primers 5′-C TGG GTG GAT TCA ACA TTG TAA AGA TCC TTT GTT G-3′, 5′-C TGG GTG GAT TCA CGC TCC ACA TTG TAA AGA TCC TTT GTT G-3′ 5′-C TGG GTG GAT TCA AGA TCC TTT GTT GTA AGT AAG ATC-3′, 5′-C TGG GTG GAT TCA GAG GCA TTT CAT TGC TCG TAG-3′ and 5′-C TGG GTG GAT TCA ATG CGG ACA TAG CAG ATT ATA AAC-3′ were used, respectively. The construction of NleF 144–189 and NleF 160–189 required the forward primers 5′-A GGC Tcc acc ATG CAT CAC TCT TCA GAG CTA TAT GG-3′ and 5′-AGGCTccaccATGTATGGTGATCTACGAGCAATG-3′, respectively, along with the ECs1815 reverse primer. The five C-terminal substitution mutants of NleF, namely NleF L186A, NleF Q187A, NleF C188A, C188S and NleF G189A were constructed using the reverse primers 5′-C TGG GTG GAT TCA TCC ACA TTG GGC AGA TCC TTT GTT G-3′, 5′-C TGG GTG GAT TCA TCC ACA TGC TAA AGA TCC TTT GTT G-3′, 5′-C TGG GTG GAT TCA TCC AGC TTG TAA AGA TCC TTT GTT G-3′, 5′-C TGG GTG GAT TCA TCC AGA TTG TAA AGA TCC TTT GTT G-3′ and 5′-C TGG GTG GAT TCA TGC ACA TTG TAA AGA TCC TTT GTT G-3′, respectively. After attachment of the attB sites in a second PCR standardized for Gateway® cloning, the resulting PCR product was shuttled in the pDONR221 entry vector, as described in the manufacturer’s instructions (Invitrogen). NleF +18 (random amino acids) was generated by removal of the native stop codon. Each construct was sequenced for verification purposes. The stability of all NleF versions was assessed by expression as renilla luciferase tagged proteins in HEK-293T cells (**Table S3**).

### Generation and Characterization of Mutant NleF Strains

The *nle*F gene of EPEC strain E2348/69 [42] was replaced using phage lambda red recombination [43] with a 75 bp scar amplified with primers NleF_F6 5′-tat cca gat ata tta gtt gta ata ata ttt atg gat tta ttt gtt aag ggg gtt ttg atG TGA AGG CTG GAG CTG CTT C-3′(upper case letters are complimentary to the cassette) and NleF_R6 5′-gac ggt cac ttt taa gaa aag gca ttt aca cag aat cct aaa cag gct cac agg cct cta aATC CTC CTT AGT TCC TAT TCC-3′. The resulting Δ*nle*F strain was complemented in single copy at the chromosomal Tn7 attachment site [44] by placement of *nle*F variants under the control of the *per* promoter [45]. Relative expression of *nle*F was determined from RNA extracted from cultures activated for 3 hours in DMEM/F12 using the TRIzol® Reagent. qRT-PCR was performed using SYBR green on a model Mx3005P thermocylcer (Stratagene) with control primers gyr_F 5′- GTCTGCGCGACAAGCGCGA-3′ and gyr_R 5′-GAAACCTGCCAGGTGAGTACC-3′ and *nle*F primers nleF_F5 5′- GGAATGACGATAGAGGATAGGGATAGTTAT-3′ and nleF_R5 5′- AGGATTAAAATCATCACTGCATCCTTCCACG-3′.

The level of activated caspase 3/7 of infected HeLa cells was assessed using flow cytometry by fluorescent inhibitor of caspases (FLICA™) labeling (Vybrant® FAM Caspase-3 and -7 Assay Kit) performed as per manufacturer’s instructions.

### Accession Numbers

The coordinates of the structures reported here have been submitted to PDB under accession number 3V3K.

## Supporting Information

Figure S1 **Interference of NleF with apoptosis signalling.** Abbreviations: TRAIL: Tumor necrosis factor Related Apoptosis Inducing Ligand, FADD: FAS-Associated protein with Death Domain, BID: BH3 interacting domain Death antagonist, tBID: trancated BID, TRAIL-R1: TRAIL Receptor 1, ER: Endoplasmic reticulum. Modified after [46].
(PDF)

Figure S2 **Interaction of Caspase-9, 8 and 4 with wild type and truncated versions of NleF.** Numbers indicate amino acids present in the deleted versions. wt, wild type (amino acid 1 to 189).
(PDF)

Figure S3 **FACS analysis of wild type NleF in HeLa cells in comparison to Protein A (negative control) and XIAP.** FACS analysis of 10,000 HeLa cells. Apoptosis was induced via TRAIL for 6 h. PI: propidium iodine, APC: allophycocyanin.
(PDF)

Figure S4 **Stereo structure of the NleF-Caspase-9 complex.**
(PDF)

Figure S5 **NleF is not a dimer.** LUMIER assay of NleF against itself and against caspase-9.
(PDF)

Figure S6 **Relative expression of nleF in cultures activated for 3 hours in DMEM/F12.** Strains complemented in single copy, ΔnleF+nleF and ΔnleF+nleF-4AA, had significantly higher levels of nleF expression than the wild type E2348/69 strain. Significance was determined using the two-tailed Wilcoxon test *p≤0.05.
(PDF)

Table S1 On/off rates and K_d_ values of the caspase-9/NleF interaction. Binding kinetics were measured five times and the average values are shown.
(DOCX)

Table S2 Data collection and refinement statistics for caspase-9 and NleF co-crystallisation.
(DOCX)

Table S3 Expression of the mutagenized NleF. N-terminally renilla luciferase-tagged NleF was expressed in HEK-293T cells and luciferase activity was measured in 10 µl HEK-293 cell lysate. The effects of NleF mutations on NleF protein levels as measured by luciferase activity are insufficient to explain the differential effects on caspase activity and apoptotic induction.
(DOCX)
