# Supplementary material for: The E. coli Effector Protein NleF Is a Caspase Inhibitor
Source: PLoS One. 2013 Mar 14;8(3):e58937. doi: 10.1371/journal.pone.0058937 (PMC3597564; doi:10.1371/journal.pone.0058937)
Supplement: Figure S1 — Interference of NleF with apoptosis signalling. Abbreviations: TRAIL: Tumor necrosis factor Related Apoptosis Inducing Ligand, FADD: FAS-Associated protein with Death Domain, BID: BH3 interacting domain Death antagonist, tBID: trancated BID, TRAIL-R1: TRAIL Receptor 1, ER: Endoplasmic reticulum. Modified after [46]. (PDF) [file pone.0058937.s001.pdf]

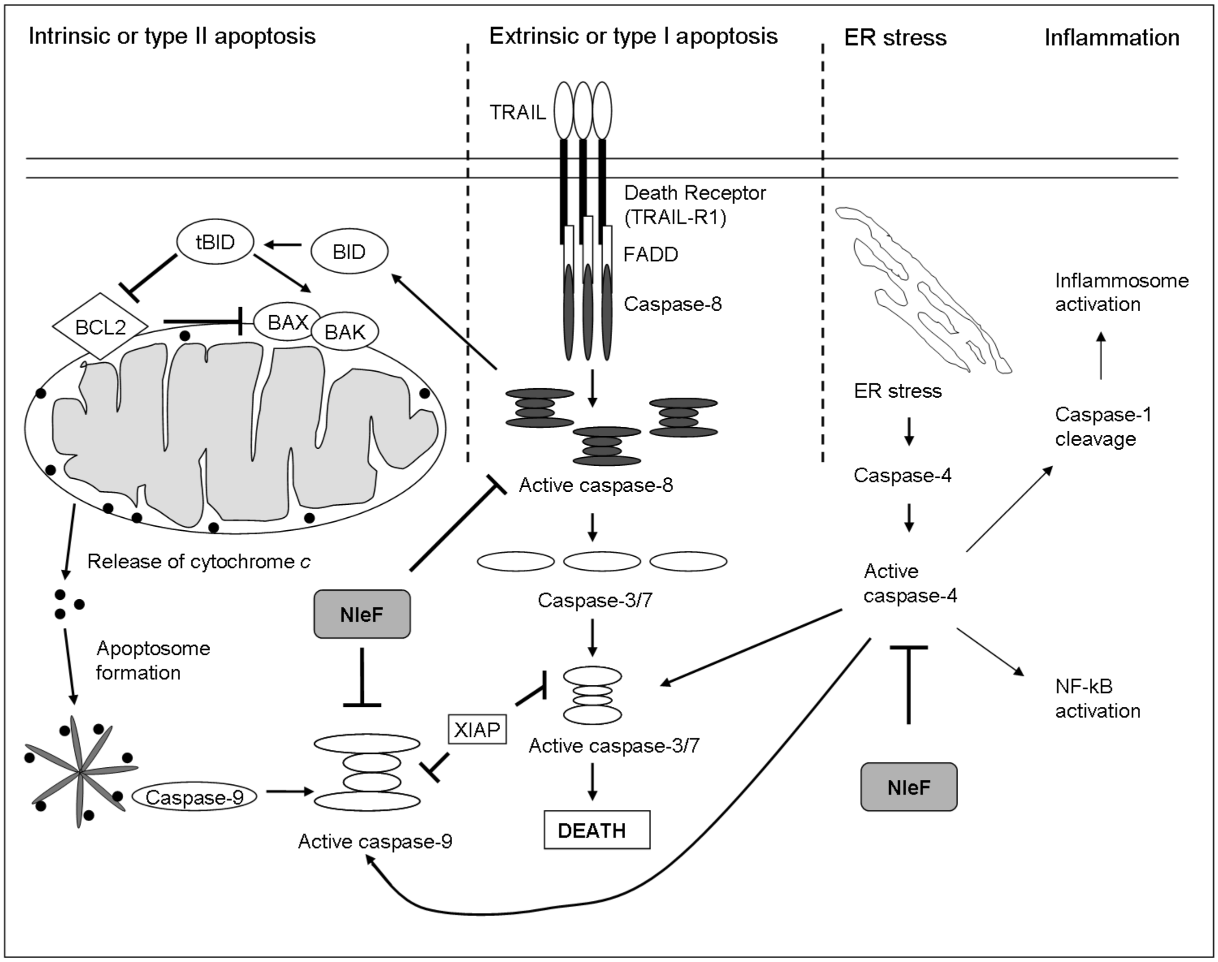

Figure S1. Interference of NleF with apoptosis signalling. Abbreviations: **TRAIL**: Tumor necrosis factor Related Apoptosis Inducing Ligand, **FADD**: FAS-Associated protein with Death Domain, **BID**: BH3 interacting domain Death antagonist, **tBID**: trancated BID, **TRAIL-R1**: TRAIL Receptor 1, **ER**: Endoplasmic reticulum. Adapted from [46].
