# Supplementary material for: The E. coli Effector Protein NleF Is a Caspase Inhibitor
Source: PLoS One. 2013 Mar 14;8(3):e58937. doi: 10.1371/journal.pone.0058937 (PMC3597564; doi:10.1371/journal.pone.0058937)
Supplement: Figure S2 — Interaction of Caspase-9, 8 and 4 with wild type and truncated versions of NleF. Numbers indicate amino acids present in the deleted versions. wt, wild type (amino acid 1 to 189). (PDF) [file pone.0058937.s002.pdf]

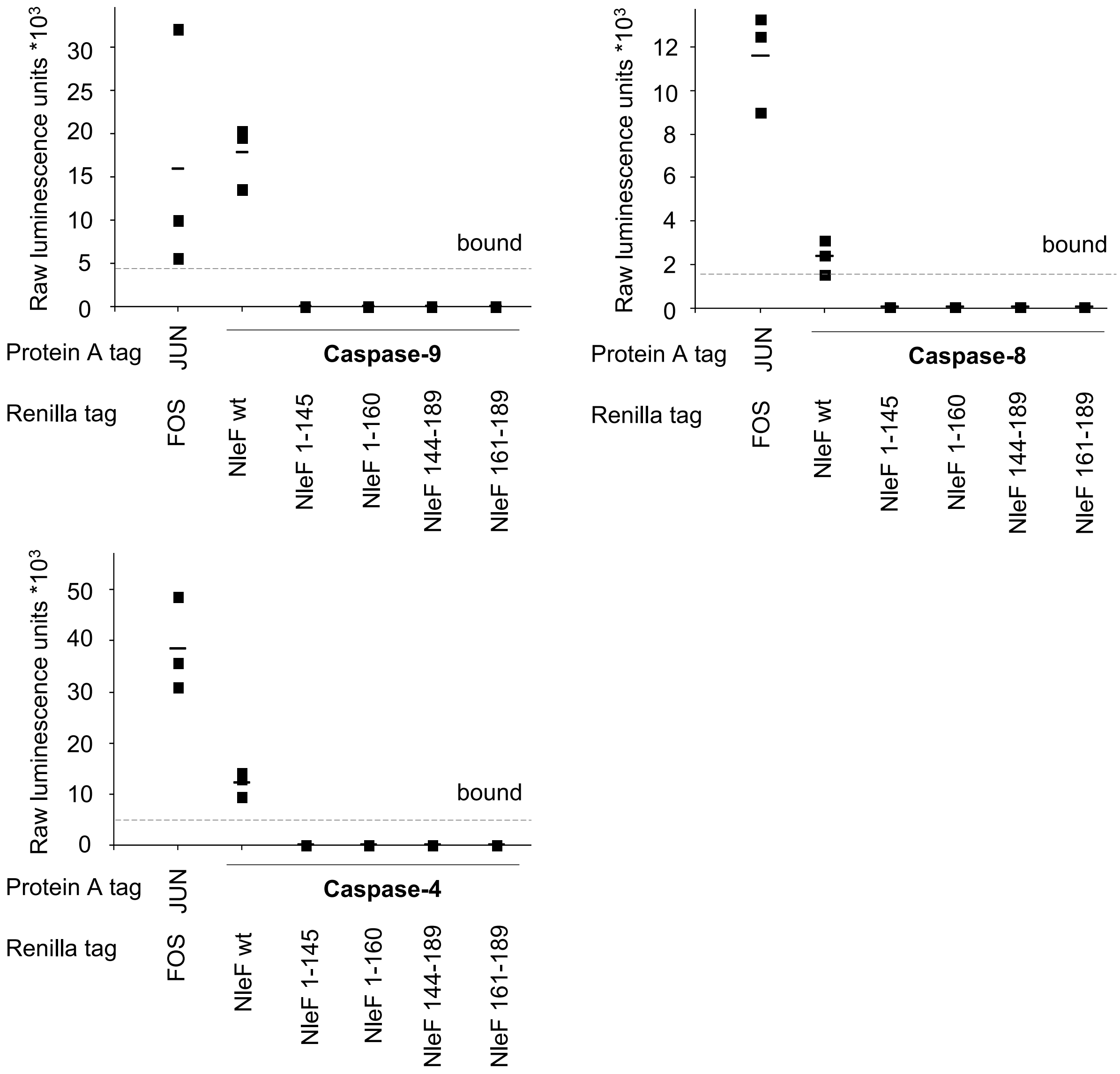

Figure S2. Interaction of Caspase-9, 8 and 4 with wild type and truncated versions of NleF. Numbers indicate amino acids present in the deleted versions. wt, wild type (amino acid 1 to 189).
