# Supplementary material for: The E. coli Effector Protein NleF Is a Caspase Inhibitor
Source: PLoS One. 2013 Mar 14;8(3):e58937. doi: 10.1371/journal.pone.0058937 (PMC3597564; doi:10.1371/journal.pone.0058937)
Supplement: Figure S3 — FACS analysis of wild type NleF in HeLa cells in comparison to Protein A (negative control) and XIAP. FACS analysis of 10,000 HeLa cells. Apoptosis was induced via TRAIL for 6 h. PI: propidium iodine, APC: allophycocyanin. (PDF) [file pone.0058937.s003.pdf]

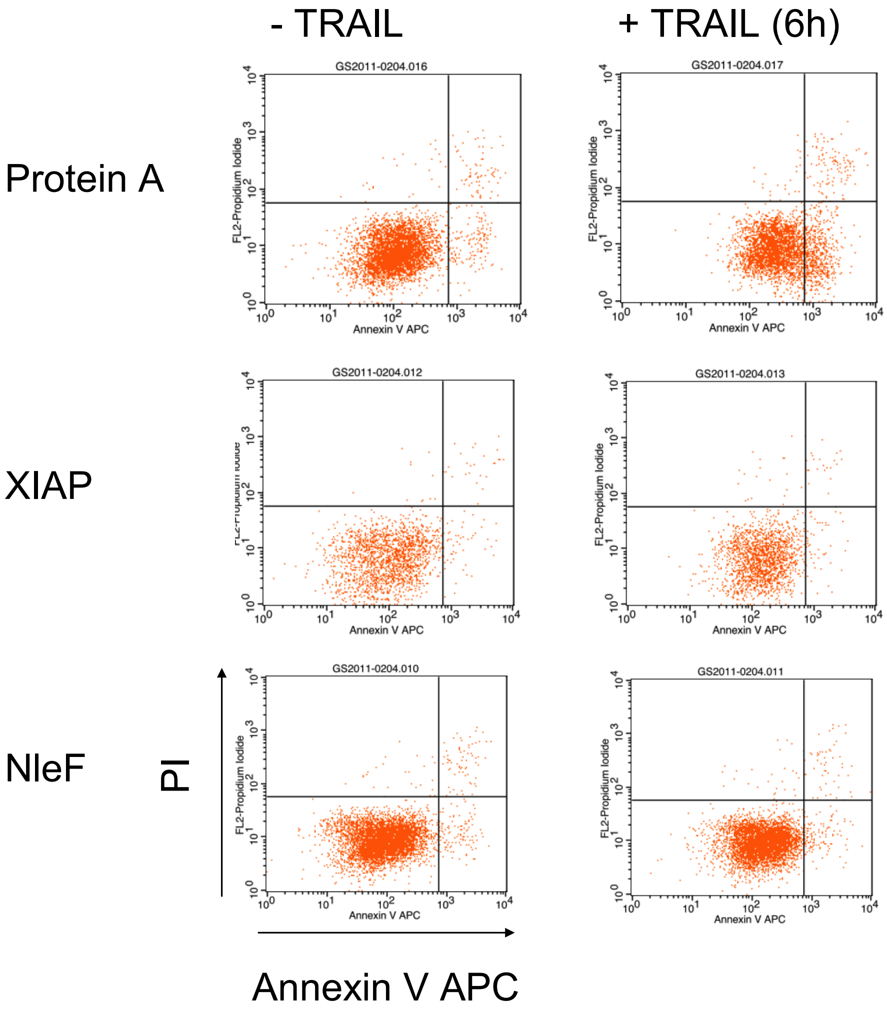

Figure S3. FACS analysis of wild type NleF in HeLa cells in comparison to Protein A (negative control) and XIAP. FACS analysis of 10,000 HeLa cells. Apoptosis was induced via TRAIL for 6h. PI: propidium iodine, APC: allophycocyanin.
