# Supplementary figures and images for: The E. coli Effector Protein NleF Is a Caspase Inhibitor
Source: PLoS One. 2013 Mar 14;8(3):e58937. doi: 10.1371/journal.pone.0058937 (PMC3597564; doi:10.1371/journal.pone.0058937)

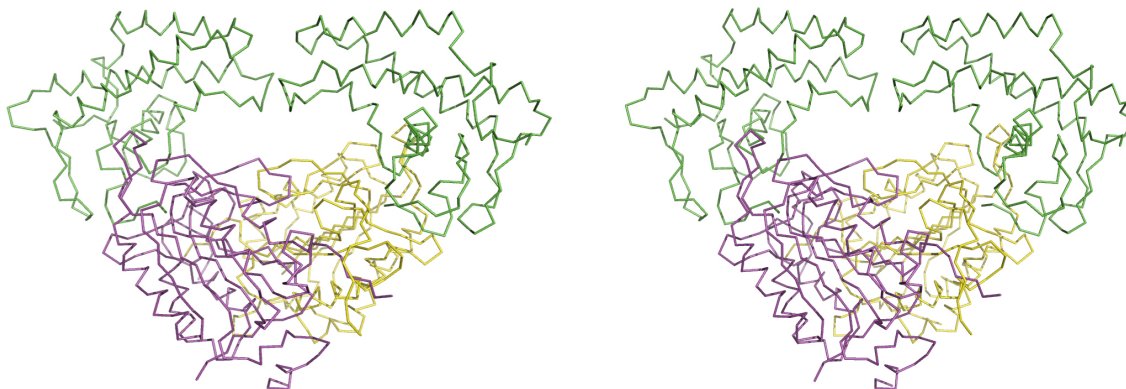

**Figure S4. Stereo structure of the NleF-Caspase-9 complex.**

Supplement: Figure S4 — Stereo structure of the NleF-Caspase-9 complex. (PDF) [file pone.0058937.s004.pdf]

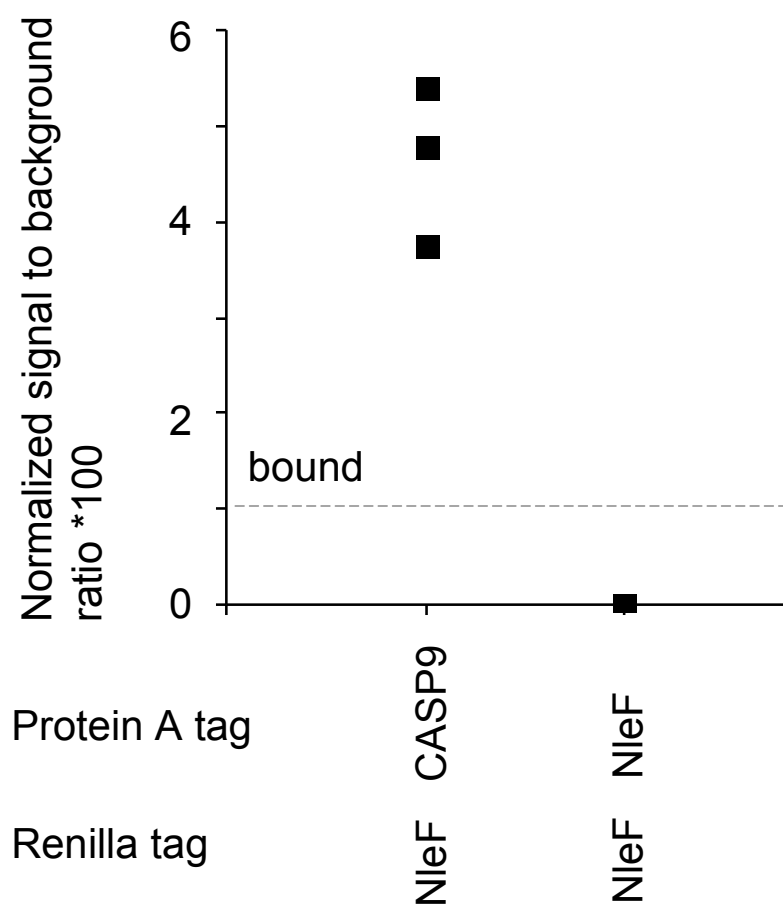

**Figure S5. NleF is not a dimer.** LUMIER assay of NleF against itself and against caspase-9.

Supplement: Figure S5 — NleF is not a dimer. LUMIER assay of NleF against itself and against caspase-9. (PDF) [file pone.0058937.s005.pdf]
