# Supplementary material for: The E. coli Effector Protein NleF Is a Caspase Inhibitor
Source: PLoS One. 2013 Mar 14;8(3):e58937. doi: 10.1371/journal.pone.0058937 (PMC3597564; doi:10.1371/journal.pone.0058937)
Supplement: Figure S6 — Relative expression of nleF in cultures activated for 3 hours in DMEM/F12. Strains complemented in single copy, ΔnleF+nleF and ΔnleF+nleF-4AA, had significantly higher levels of nleF expression than the wild type E2348/69 strain. Significance was determined using the two-tailed Wilcoxon test *p≤0.05. (PDF) [file pone.0058937.s006.pdf]

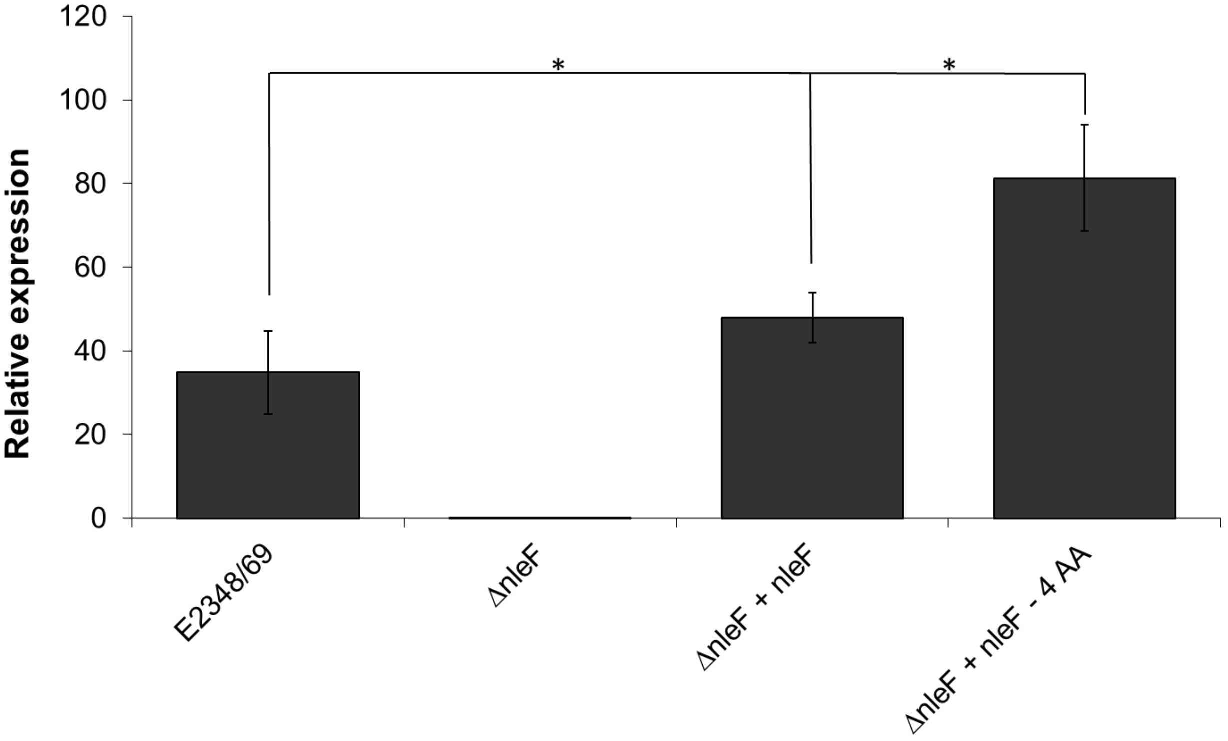

Figure S6: Relative expression of *nle*F in cultures activated for 3 hours in DMEM/F12. Strains complemented in single copy, Δ*nle*F+*nle*F and Δ*nle*F+*nle*F-4AA, had significantly higher levels of *nleF* expression than the wild type E2348/69 strain. Significance was determined using the two-tailed Wilcoxon test * $p \leq 0.05$.
