# Supplementary material for: The E. coli Effector Protein NleF Is a Caspase Inhibitor
Source: PLoS One. 2013 Mar 14;8(3):e58937. doi: 10.1371/journal.pone.0058937 (PMC3597564; doi:10.1371/journal.pone.0058937)
Supplement: Table S1 — On/off rates and Kd values of the caspase-9/NleF interaction. Binding kinetics were measured five times and the average values are shown. (DOCX) [file pone.0058937.s007.docx]

**Table S1: On/off rates and K_d_ values of the caspase‑9/NleF interaction.** Binding kinetics were measured five times and the average values are shown.

| **Sam5 blue** | **K_on_ [nM^-1^ s^-1^]** | **K_off_ [s^-1^]** | **K_d_ [nM]** |
| --- | --- | --- | --- |
| Chip1 [1] | 0.000020 ± 0.0 | 0.001109 ± 0.000136 | 55.0 ± 8.0 |
| Chip1 [2] | 0.000009 ± 0.0 | 0.000256 ± 0.000011 | 27.9 ± 1.6 |
| Chip1 [3] | 0.000016 ± 0.000002 | 0.000853 ± 0.000287 | 54.6 ± 24.7 |
| Chip2 [1] | 0.000011 ± 0.000002 | 0.000336 ± 0.000295 | 29.8 ± 5.0 |
| Chip3 [2] | 0.000024 ± 0.000002 | 0.000618 ± 0.000172 | 26.3 ± 9.3 |
| Mean | 0.000016 | 0.000634 | 38.7 |
