# Supplementary material for: The E. coli Effector Protein NleF Is a Caspase Inhibitor
Source: PLoS One. 2013 Mar 14;8(3):e58937. doi: 10.1371/journal.pone.0058937 (PMC3597564; doi:10.1371/journal.pone.0058937)
Supplement: Table S2 — Data collection and refinement statistics for caspase-9 and NleF co-crystallisation. (DOCX) [file pone.0058937.s008.docx]

**Table S2.** Data collection and refinement statistics for caspase‑9 and NleF co-crystallisation.

|  | Caspase-9-NleF |
| --- | --- |
| **Data collection and Processing**  No. of crystals used  Wavelength [Å]  Space group  Unit cell parameters  a, b, c [Å]  α,β,γ   Matthews coefficient [Å³/Da]  Solvent content [%] Diffraction data Resolution [Å]  Unique reflections  R(I)_sym_ [%]  Completeness [%]  Redundancy  I/σ(I)  **Refinement**  Resolution range [Å]  Reflections used in refinement (work/free)  Final R values for all reflections (work/free) [%]  Protein atoms RMSDs Bonds [Å]  Angles [°] Ramachandran plot Residues in most favoured regions [%]  Residues in additional allowed regions [%]  Residues in generously allowed regions [%]  Residues in disallowed regions [%]  **Mean B factor [Å^2^]**  Protein | 1  1.0015  P2_1_2_1_2_1_  100.7, 209.91, 317.23  90.0, 90.0, 90.0  3.3  62.7  3.49 (3.77-3.49)  84,797 (17,178)  12.6 (58.1)  98.8 (98.3)  3.3 (3.4)  8.3 (2.0)  174.08 – 3.49  83 041 / 1 689  22.5 / 26.0  25,500  0.009  1.06  86.5  12.6  0.2  0.7  86.4 |
