# Supplementary material for: The E. coli Effector Protein NleF Is a Caspase Inhibitor
Source: PLoS One. 2013 Mar 14;8(3):e58937. doi: 10.1371/journal.pone.0058937 (PMC3597564; doi:10.1371/journal.pone.0058937)
Supplement: Table S3 — Expression of the mutagenized NleF. N-terminally renilla luciferase-tagged NleF was expressed in HEK-293T cells and luciferase activity was measured in 10 µl HEK-293 cell lysate. The effects of NleF mutations on NleF protein levels as measured by luciferase activity are insufficient to explain the differential effects on caspase activity and apoptotic induction. (DOCX) [file pone.0058937.s009.docx]

**Table S3. Expression of the mutagenized NleF.** N-terminally renilla luciferase**-**tagged NleF was expressed in HEK-293T cells and luciferase activity was measured in 10 µl HEK-293 cell lysate. The effects of NleF mutations on NleF protein levels as measured by luciferase activity are insufficient to explain the differential effects on caspase activity and apoptotic induction.

| **NleF construct** | **Luciferase activity** |
| --- | --- |
| NleF +1 | 86,046 |
| NleF -1 | 189,843 |
| NleF -4 | 102,746 |
| NleF L186A | 157,490 |
| NleF Q187A | 127,316 |
| NleF C188A | 133,733 |
| NleF C188S | 131,229 |
| NleF G189A | 172,160 |
| NleF +18 | 63,443 |
| NleF aa 1-160 | 33,096 |
| NleF aa 1-145 | 13,840 |
| NleF aa 144-189 | 44,752 |
| NleF aa 161-189 | 83,766 |
| NleF wild type | 30,382 |
